# Flourishing in university students: cross-sectional associations with personal and social FoMO, loneliness, and belonging

**DOI:** 10.3389/fnbeh.2026.1914187

**Published:** 2026-09-29

**Authors:** Osman Akay

**Affiliations:** 1 Department of Social Work, Seydikemer School of Applied Sciences, Muğla Sıtkı Koçman University, Muğla, Türkiye; 2 Department of Social Work, Faculty of Health Sciences, Istanbul Medipol University, Istanbul, Türkiye

**Keywords:** Fear of Missing Out (FoMO), flourishing, loneliness, sense of belonging, university students

## Abstract

Fear of missing out (FoMO), loneliness, and belonging concern different aspects of students’ relational experience, but their shared and unique cross-sectional associations with flourishing remain unclear. This study examined these associations in university students in Türkiye while distinguishing Personal from Social FoMO and acceptance/inclusion from rejection/exclusion. An online cross-sectional survey was completed by 499 students aged 18–30 years (M = 22.13, SD = 3.23; 76.4% female). Factor structure and internal consistency were examined before Pearson correlations, hierarchical regression with HC3 robust standard errors, relative-importance analysis, and sensitivity analyses were conducted. At the zero-order level, both FoMO facets and loneliness were negatively associated with flourishing, whereas acceptance/inclusion and reverse-scored rejection/exclusion were positively associated with flourishing (all *p* < 0.001). The final seven-predictor model explained 51.2% of flourishing variance. Loneliness showed a small negative unique association (*β* = −0.136, *p* = 0.016), acceptance/inclusion the largest positive association (*β* = 0.526, *p* < 0.001), and reverse-scored rejection/exclusion a smaller positive association (*β* = 0.138, *p* = 0.023); neither FoMO facet had a significant unique coefficient. Acceptance/inclusion and loneliness remained significant after explicitly relational Flourishing Scale items were removed, whereas the rejection/exclusion coefficient did not. These findings describe concurrent associations rather than a causal sequence, and they point to no specific intervention target. The cross-sectional self-report design, convenience and snowball sampling, and female-predominant sample limit causal inference and generalizability.

## Introduction

### University flourishing in a digital and relational context

University life is not only an academic setting but also a social context in which students form interpersonal networks and develop a sense of belonging (Holzer et al., 2021; Pleschová et al., 2026). The present study therefore approaches students’ positive functioning through flourishing rather than treating the absence of distress as its outcome. Flourishing is operationalized as a broad self-appraisal of psychosocial functioning, reflected in meaning and purpose, supportive relationships, engagement, contribution to others, competence, positive self-regard, optimism, and social respect (Diener et al., 2010). The term flourishing is used throughout because it reflects the scale’s broad psychosocial content more accurately than the narrower label psychological well-being.

The social context in which students pursue these goals is increasingly digital. In 2024, 97% of people aged 16–29 years in the European Union used the internet daily, and 88% participated in online social-network activities (Eurostat, 2025). Data collected in 2022 through the first EU-wide Loneliness Survey indicated that, depending on the measure, 36–40% of respondents experienced at least occasional loneliness and that loneliness prevalence declined with age (Berlingieri et al., 2023). These figures do not establish that digital engagement is uniformly harmful. Analyses of the same survey showed that spending more than 2 h per day on social-networking sites and intensive passive social-media use were associated with a greater prevalence of loneliness, whereas intensive instant-messaging and active social-media use were not significantly associated with loneliness (d’Hombres et al., 2024). These findings are used only to situate the broader digital context; the present study did not measure these specific usage patterns. Instead, it examines the concurrent associations of digital-social concerns, indexed by Personal and Social FoMO, with loneliness, belonging, and flourishing.

### Theoretical framework

Self-determination theory provides the organizing framework for the study. It proposes that well-being is supported by the satisfaction of three basic psychological needs: autonomy, competence, and relatedness (Deci and Ryan, 2000). Relatedness concerns feeling connected to and cared for by others while also being able to care for them. The belongingness hypothesis similarly conceptualizes the formation and maintenance of stable, positive interpersonal bonds as a fundamental human motivation (Baumeister and Leary, 1995). Taken together, these perspectives provide a rationale for treating FoMO, loneliness, and belonging as distinct but related aspects of a broader relational domain. FoMO concerns apprehension about missing personally valued opportunities or experiences enjoyed by others (Przybylski et al., 2013; Zhang et al., 2020). Loneliness refers to the aversive subjective perception that the quantity or quality of one’s relationships is insufficient (Berlingieri et al., 2023; Vitale and Smith, 2022), whereas belonging reflects a more general sense of acceptance and inclusion versus rejection and exclusion (Malone et al., 2012).

This distinction matters for both theory and analysis. FoMO involves concern about missed opportunities and others’ ongoing activities, but it does not, by definition, indicate that a person is currently lonely or excluded. Loneliness may occur despite frequent social interaction when relationships are perceived as insufficient in quality or quantity, whereas belonging captures a more general sense of being accepted, included, and connected. The constructs may therefore share variance while retaining different associations with flourishing. They are treated here as concurrent correlates rather than as successive stages of a causal process. Evidence synthesized across human and animal research further indicates that social affiliation engages reward-related systems and can buffer stress, whereas human loneliness is associated with altered stress responsivity and social-information processing (Vitale and Smith, 2022).

### FoMO and flourishing

FoMO was initially defined as a pervasive apprehension that others may be having rewarding experiences from which one is absent (Przybylski et al., 2013). The self-concept formulation used in the present study distinguishes Personal FoMO, concerning missed experiences associated with the private self, from Social FoMO, concerning experiences enjoyed by others and relevant to the public or social self (Zhang et al., 2020). This distinction avoids assuming that all missed opportunities have the same psychological meaning. University research has associated FoMO with adverse aspects of daily functioning during digital engagement and with problematic social-media and smartphone use (Hayran and Anik, 2021; O'Brien et al., 2023). However, a three-wave study found that the associations of FoMO with psychological need satisfaction and well-being were primarily between-person, with only limited evidence of corresponding within-person processes (Groenestein et al., 2025). The present analysis therefore examines the concurrent associations of Personal and Social FoMO with flourishing without assigning temporal precedence to FoMO.

### Loneliness, belonging, and flourishing

Loneliness represents the subjective perception that the quantity or quality of one’s relationships is insufficient, rather than objective social isolation alone (Berlingieri et al., 2023). In Turkish university samples, loneliness has been negatively associated with psychological well-being and positively associated with social-media addiction (Cinalioğlu and Gazioğlu, 2022; Uyaroğlu et al., 2022). Longitudinal evidence also cautions against a single directional account. A three-wave panel study found that stable between-person differences in FoMO and loneliness were related, while the within-person path was observed from loneliness to subsequent FoMO rather than from FoMO to subsequent loneliness (Bonfanti et al., 2023). More recently, a two-wave study with a 12-month interval found that baseline loneliness was positively associated with later trait- and state-FoMO among college students (Wang et al., 2025). Taken together, these findings do not support a uniform temporal sequence in which FoMO precedes loneliness. A nationwide cross-sectional study of Danish high-school students similarly distinguished contextual social disconnectedness from subjective loneliness and found that both were associated with poorer mental well-being (Santini et al., 2021).

Belonging refers to a generalized experience of acceptance and inclusion and the absence of rejection or exclusion (Malone et al., 2012). It is particularly relevant to university life, where students develop peer networks and a sense of institutional membership. Recent higher-education research links belonging with greater well-being and lower loneliness. Across five European universities, belonging was positively associated with well-being and negatively associated with loneliness, although the strength of the association with loneliness varied across institutions (Pleschová et al., 2026). A meta-analysis of 56 higher-education studies reported a moderate-to-strong negative association between belonging and loneliness, r=−.48 , although between-study heterogeneity was substantial (Dost, 2026). In a UK university sample, both collegiate-community belonging and degree-department belonging were negatively associated with loneliness (Dost, 2025). These findings support examining belonging and loneliness as related but non-equivalent constructs.

### Construct distinctiveness

A methodological complication is that the Flourishing Scale contains explicitly relational content, including supportive and rewarding relationships, contributing to others’ happiness and well-being, and being respected by others (Diener et al., 2010). A strong association between belonging and flourishing could therefore reflect both substantive relatedness and item-content overlap. Reliability coefficients alone cannot resolve this issue. The analysis consequently evaluates the factorial distinctiveness of belonging and flourishing and repeats the primary regression after excluding the explicitly relational Flourishing Scale items. The factorial analyses assess whether the constructs are empirically separable, whereas the item-omission analysis examines the extent to which their observed association depends on shared relational content. An association that remains after these items are excluded is interpreted as evidence that the finding is not fully attributable to explicit item overlap, rather than as proof of discriminant validity or causal priority. In a related sensitivity analysis, Lu et al. (2024) similarly found that perceived social support remained associated with psychological well-being after 14 relationship-related items were removed from the outcome measure.

### The present study

Prior studies have often examined FoMO, loneliness, belonging, or well-being in separate models, used an undifferentiated FoMO total score, or emphasized digital-use mechanisms that were not directly measured. Few studies have examined Personal and Social FoMO, loneliness, the two belonging facets, and flourishing within the same model. This study evaluates their shared and unique associations in a Turkish university sample. Türkiye provides a useful regional context, but cultural orientation was not measured; collectivism and self-construal are therefore not used to explain the findings.

The analytic model was organized into conceptually ordered blocks. The hierarchical model entered age and gender in Block 1, Personal and Social FoMO in Block 2, loneliness in Block 3, and the two belonging facets in Block 4. The block order was used to estimate incremental variance rather than temporal sequence. Semipartial correlations and LMG relative importance were used to examine shared predictor variance, and the indirect-association models were exploratory.

### Conceptual and analytic roles of the study variables

**Table:** 

Construct	Conceptual role	Primary analytic role
Age and gender	Background characteristics; not mechanisms	Block 1 covariates
Personal and social FoMO	Concern about missed personal rewards or social standing	Block 2 focal correlates; facets analyzed separately
Loneliness	Perceived deficit in desired relationships	Block 3 focal correlate
Acceptance/inclusion and rejection/exclusion	General appraisal of achieved belonging	Block 4 focal correlates; facets analyzed separately
Flourishing	Meaning, competence, engagement, optimism, self-regard, and relationships	Outcome; relational-item sensitivity analysis

### Aims and hypotheses

The primary aim of this study was to examine the zero-order and mutually adjusted cross-sectional associations among FoMO, loneliness, belonging, and flourishing. Three directional hypotheses were proposed:

*H1*: FoMO would be positively associated with loneliness and negatively associated with belonging.

*H2*: Loneliness would be negatively associated with flourishing, whereas belonging would be positively associated with flourishing.

*H3*: Loneliness and belonging would show unique associations with flourishing after adjustment for age, gender, and FoMO.

At the operational level, these construct-level hypotheses were evaluated using Personal and Social FoMO and the acceptance/inclusion and reverse-scored rejection/exclusion facets of belonging. Incremental explained variance, relative importance, measurement overlap, sensitivity analyses, and cross-sectional indirect associations were examined to provide a more detailed account of shared and unique associations.

## Methods

### Design

This study used a cross-sectional online self-report design to examine concurrent associations among Personal and Social FoMO, loneliness, belonging, and flourishing in university students in Türkiye. Because all variables were measured at one time point, the analyses cannot establish temporal ordering, causal effects, or underlying psychological mechanisms.

Reporting followed the STROBE checklist for cross-sectional studies (von Elm et al., 2007); which is provided as Supplementary material.

### Participants and procedure

Data were collected online between 1 and 5 June 2026 using convenience and snowball sampling. The initial survey announcement was circulated among students in the Department of Social Work at Istanbul Medipol University and was subsequently redistributed through participants’ online networks. Participation was not restricted to Istanbul Medipol University, social work programmes, or a particular year of study. Institutional and departmental affiliations were not collected; therefore, the number and identity of participating institutions are unknown. Recruitment-chain identifiers and referral links between participants were not retained; therefore, the number, size, and composition of the recruitment chains could not be determined. Eligibility was limited to individuals who self-identified as current university students and were aged 18–30 years; no additional participant-level eligibility criteria were applied. A total of 504 survey records were submitted. Data-quality screening examined missingness, invariant response patterns, and exact response duplication. Three records were excluded because the same lowest response option was selected across all 35 substantive scale items, representing an extreme straightlining pattern. This left 501 records, none of which contained missing values in the primary analytic variables. Three of these records displayed an exact response pattern across demographic variables, contextual variables, and all 35 scale items. Because the data did not permit confirmation that these records represented the same individual, they were treated as exact duplicate response patterns rather than confirmed duplicate participants. The earliest record was retained and the two later records were excluded from the primary analyses, yielding an analytic sample of 499. All principal analyses were repeated with all 501 records retained, and the substantive pattern of findings was unchanged.

The primary analytic sample had a mean age of 22.13 years (SD = 3.23, range = 18–30). Of the 499 participants, 381 identified as female (76.4%) and 118 as male (23.6%). For regression analyses, gender was represented by a binary indicator coded 0 = male and 1 = female. The female-predominant composition should be considered when generalizing the findings to male students.

An *a priori* sample-size planning analysis was conducted using G*Power 3.1 before data collection and was documented in the ethics application. The planned analysis was a fixed-model multiple regression based on an increment in R^2^, with an alpha level of 0.05, statistical power of 0.80, and a medium effect size of f^2^ = 0.15. The initially planned model included five predictors: age, gender, total FoMO, loneliness, and total belonging, with the three substantive constructs treated as the tested predictor set. Under this specification, G*Power yielded a minimum sample size of 77. The ethics protocol specified a recruitment target of at least 400 participants, and the final analytic sample of 499 exceeded both values.

### Ethical approval

Ethical approval was granted by the Istanbul Medipol University Social Sciences Scientific Research Ethics Committee (Decision No. 501, dated 1 June 2026). Data collection was conducted online from 1 to 5 June 2026 in accordance with the principles of the Declaration of Helsinki. Before accessing the questionnaire, participants viewed an electronic information and consent statement explaining the scientific purpose of the study, the voluntary nature of participation, their right to discontinue participation at any time without penalty, and the restriction of the collected information to scientific use. Only individuals who electronically indicated their voluntary consent were permitted to proceed to the questionnaire. No participation incentive was provided.

## Measures

### Fear of missing out

FoMO was assessed using the Turkish social-media adaptation by Çelik and Özkara (2022) of the Personal and Social FoMO framework developed by Zhang et al. (2020). The adapted instrument comprised nine items rated from 1 (strongly disagree) to 7 (strongly agree): five Personal FoMO items and four Social FoMO items. Higher mean scores indicate greater FoMO within each domain. The primary analyses used the Personal and Social FoMO subscale scores rather than a nine-item total score. In the present sample, the correlated two-factor model provided a substantially better representation of the item covariance structure than the one-factor model, although the fit of the two-factor model was not uniformly satisfactory, particularly with respect to RMSEA.

### Loneliness

Loneliness was assessed using the six-item short form of the UCLA Loneliness Scale (ULS-6), developed by Neto (2014) and adapted into Turkish by Sarıçam (2023). Items were rated on a five-point Likert-type scale and averaged, with higher scores indicating greater perceived loneliness. Sarıçam (2023) reported a Cronbach’s alpha of 0.77 for the Turkish version. In the present primary analytic sample, internal consistency was high (Cronbach’s *α* = 0.899; McDonald’s *ω* = 0.926).

### Belonging

Belonging was assessed with the 12-item General Belongingness Scale developed by Malone et al. (2012) and adapted into Turkish for university students by Duru (2015). Items were rated from 1 (strongly disagree) to 7 (strongly agree). The first six items represented acceptance/inclusion, and the final six represented rejection/exclusion and were reverse scored so that higher values indicated less rejection/exclusion. The acceptance/inclusion and reverse-scored rejection/exclusion facet means were analyzed separately. In the present sample, the correlated two-factor model fit substantially better than the one-factor alternative, although its overall fit remained mixed. A single total belonging score was therefore not used in the primary regression.

### Flourishing

Flourishing was assessed with the eight-item Flourishing Scale developed by Diener et al. (2010) and adapted into Turkish by Telef (2013) under the title Psychological Well-Being Scale. Response options ranged from 1 (strongly disagree) to 7 (strongly agree). Items were averaged, with higher scores indicating greater flourishing.

### Demographic and contextual variables

Age and gender were included as background covariates. Gender was represented by a binary indicator coded 0 = male and 1 = female in the regression analyses. Other categorical variables were used only to describe the sample and were not entered into the primary model. Additional items assessed marital status, year in school, perceived income, daily social-media duration, checking frequency, prior mental-health consultation, psychiatric diagnosis, current psychological support, psychiatric medication, general health, sleep duration, and physical activity. These variables were not entered indiscriminately into the primary model because no *a priori* confounding role had been specified and several variables could plausibly be correlates, consequences, or mediators of the focal constructs.

## Data analysis

Analyses were performed in Python 3.12.13 using pandas 2.2.3, NumPy 2.3.5, and SciPy 1.17.0, and in R 4.6.0 under webR using lavaan 0.7–2.3166. Data screening examined missingness, extreme straightlining across all 35 substantive scale items, and exact response duplication across demographic, contextual, and scale variables. Scale scores were calculated as item means. Cronbach’s alpha and McDonald’s omega were used to evaluate internal consistency.

Because the items were ordinal, confirmatory factor analyses were estimated in lavaan with the WLSMV specification, theta parameterization, standardized latent variances, and the items declared ordered. Lavaan implements this specification using diagonally weighted least squares with a scaled-shifted test. One- and two-factor models were compared for FoMO and belonging; one-factor models were examined for loneliness and flourishing; and five- and six-factor combined measurement models were compared. Model fit was evaluated jointly using robust or scaled comparative fit index (CFI), Tucker-Lewis index (TLI), root mean square error of approximation (RMSEA) with its 95% confidence interval, and standardized root mean square residual (SRMR), without treating any single threshold as decisive. The factorial distinctiveness of belonging and flourishing was examined using heterotrait-monotrait ratios of correlations (HTMT; Henseler et al., 2015), latent correlations, and alternative models specifying acceptance/inclusion, reverse-scored rejection/exclusion, and flourishing as separate or merged factors. For the combined six-factor measurement model, standardized factor loadings and indicator *R*^2^ values were inspected, and local misfit was evaluated using modification indices and raw residual correlations calculated as the observed polychoric correlations minus the model-implied correlations. These diagnostics were used descriptively to identify the location of misfit; no post-hoc cross-loadings or residual covariances were added because such modifications were not prespecified and could capitalize on sample-specific associations.

Zero-order associations were estimated with Pearson correlations and 95% confidence intervals based on Fisher’s *z* transformation. Spearman correlations were examined as a rank-based sensitivity analysis. Differences between the Personal and Social FoMO correlations with the same outcome were examined using tests for dependent, overlapping correlations.

The primary outcome model was a four-block hierarchical regression. Age was entered as a continuous variable, and gender was represented by a binary indicator coded 0 = male and 1 = female. Age and gender were entered in Block 1, Personal and Social FoMO in Block 2, loneliness in Block 3, and acceptance/inclusion and reverse-scored rejection/exclusion in Block 4. The block order provided a theory-informed framework for estimating incremental explained variance and was not interpreted as a temporal, causal, or mechanistic sequence. The final model reported unstandardized and standardized coefficients, 95% confidence intervals, partial and semipartial correlations, tolerance, and variance inflation factors (VIFs). Because the Breusch–Pagan test indicated heteroskedasticity, all confidence intervals and *p* values for the regression coefficients were based on HC3 heteroskedasticity-consistent covariance estimates (MacKinnon and White, 1985). The joint contribution of the variables added at each hierarchical block was evaluated using an HC3-robust Wald *F* test of the newly entered coefficients in the corresponding expanded model. Changes in *R*^2^ were treated as descriptive increments in explained variance; conventional homoskedastic ΔF tests were not used for statistical inference. Linearity, residual distribution, leverage, studentized residuals, and Cook’s distance were also examined. LMG relative importance analysis was used to partition model *R*^2^ across correlated predictors without depending on a single entry order.

To address potential criterion contamination, a sensitivity outcome omitted the explicitly relational Flourishing Scale items 2, 4, and 8. Additional sensitivity models retained all 501 records, restricted the sample to ages 18–29, and omitted observations exceeding the Cook’s D screening threshold of 4/N. The latter screen was not used to redefine the primary sample because statistical influence flags do not by themselves demonstrate invalid data.

Finally, exploratory six-factor latent direct- and indirect-association models were estimated. Both models retained the same six-factor item-level measurement specification used in the combined CFA: Personal FoMO, Social FoMO, loneliness, acceptance/inclusion, reverse-scored rejection/exclusion, and flourishing. Age and gender were included as observed background covariates. The indirect-association model estimated paths from Personal and Social FoMO to flourishing through loneliness and the two belonging facets. Residual covariances among the three concurrent intervening variables were freely estimated because they were not assumed to be conditionally independent. Standardized delta-method confidence intervals were obtained from lavaan. A parallel observed-score model with 5,000 percentile bootstrap samples was examined as a sensitivity analysis. The reporting of the latent indirect-association model included all constituent standardized structural paths, covariate paths, residual correlations among the concurrent intervening variables, and *R*^2^ values for the endogenous latent variables. A path diagram was used to display the focal standardized coefficients, while the complete covariate coefficients were reported in the accompanying table to preserve visual clarity. The fit indices of the latent structural models were not treated as directly comparable with those of the stand-alone six-factor CFA. Adding age and gender as fixed observed covariates introduced item-covariate moments, changed the model degrees of freedom and the baseline model used in incremental fit indices, and therefore could improve global CFI, TLI, and RMSEA values without resolving item-level measurement misfit. Structural-model fit was consequently interpreted within each conditional specification and was not used to override the mixed fit of the stand-alone measurement models. The indirect estimates were interpreted as cross-sectional statistical associations rather than evidence of causal mediation or temporal ordering. The study and analysis plan were not preregistered. For interpretive clarity, zero-order correlations and hierarchical regression were treated as the primary analyses. Confirmatory factor analyses, factorial-distinctiveness tests, dependent-correlation comparisons, and LMG relative-importance analysis were treated as complementary analyses. Models retaining all 501 records, restricting the age range, excluding explicitly relational Flourishing Scale items, and screening observations exceeding the Cook’s distance threshold were treated as sensitivity analyses. The latent direct- and indirect-association models were treated as exploratory. The study was not preregistered. Zero-order correlations and hierarchical regression were treated as the primary analyses. CFA and relative-importance analyses were complementary; record-retention, age-restriction, item-omission, and influence-screening models were sensitivity analyses; and the latent structural models were exploratory.

## Results

### Data screening and sample characteristics

Of the 504 submitted survey records, three were excluded because they showed extreme straightlining across all 35 substantive scale items. Among the remaining 501 records, three displayed an exact response pattern across demographic, contextual, and scale variables. The earliest of these records was retained and the two later records were excluded, yielding a primary analytic sample of 499. No missing values were present in the primary analytic variables. Retaining all 501 records produced the same substantive pattern of findings. Participants in the primary sample were aged 18–30 years (M = 22.13, SD = 3.23), and 76.4% were female. The gender imbalance limits generalization to male students.

No participant selected the response category of more than 5 h of daily social-media use. In contrast, 163 participants (32.7%) reported checking social media constantly. These items assess different dimensions—estimated cumulative duration and checking frequency—and their response-category boundaries are reported explicitly in Table 1. The apparent contrast should not be treated as a data error, but the self-reported duration category warrants cautious interpretation. The principal descriptive and analytical results are presented in Tables 2–7.

**Table 1:** Participant characteristics.

Variable/category	*n* or M (SD)	% or range
Age (years)	22.13 (3.23)	18–30
Gender		
Female	381	76.4
Male	118	23.6
Marital status		
Single	436	87.4
Married	49	9.8
Divorced	3	0.6
Other	11	2.2
Year in school		
1st year	85	17.0
2nd year	147	29.5
3rd year	69	13.8
4th year	33	6.6
5th year	74	14.8
6th year	91	18.2
Perceived income level		
Low	71	14.2
Middle	375	75.2
High	53	10.6
Daily social media use		
Less than 1 h	129	25.9
1–3 h	222	44.5
3–5 h	148	29.7
More than 5 h	0	0.0
Social media checking frequency		
Rarely	28	5.6
A few times a day	131	26.3
A few times an hour	177	35.5
Constantly	163	32.7
Prior mental health consultation		
Yes	93	18.6
No	406	81.4
Psychiatric diagnosis		
Yes	34	6.8
No	452	90.6
Prefer not to say	13	2.6
Currently receiving psychological support		
Yes	24	4.8
No	475	95.2
Regular psychiatric medication use		
Yes	16	3.2
No	483	96.8
General health status		
Very poor	8	1.6
Poor	9	1.8
Fair	157	31.5
Good	277	55.5
Very good	48	9.6
Daily sleep duration		
Less than 5 h	27	5.4
5–6 h	116	23.2
6–8 h	284	56.9
More than 8 h	72	14.4
Regular physical activity		
Yes	165	33.1
No	334	66.9

*N =* 499. Of 504 submitted records, three extreme straightlining records and two later records from an exact three-record response pattern were excluded. No missing values were present in the primary variables.

**Table 2:** Descriptive statistics and internal consistency of primary constructs.

Construct	Items	Range	M	SD	*α*	*ω*
Personal FoMO	5	1–7	2.394	1.308	0.909	0.945
Social FoMO	4	1–7	1.876	1.077	0.912	0.951
Loneliness	6	1–5	2.353	0.846	0.899	0.926
Acceptance/inclusion	6	1–7	5.448	1.177	0.895	0.924
Rejection/exclusion (reversed)	6	1–7	5.431	1.356	0.899	0.937
Flourishing	8	1–7	5.303	1.076	0.891	0.918

*N* = 499. Scores are item means. Higher reverse-scored rejection/exclusion values indicate less rejection/exclusion.

**Table 3:** Pearson correlations among demographics and primary constructs.

Variable	1	2	3	4	5	6	7	8
1. Age	-							
2. Female	−0.137	-						
3. Personal FoMO	−0.186	0.234	-					
4. Social FoMO	−0.189	0.128	0.581	-				
5. Loneliness	−0.125	0.104	0.318	0.302	-			
6. Acceptance	0.129	−0.017	−0.222	−0.328	−0.407	-		
7. Rejection (rev.)	0.124	−0.053	−0.336	−0.418	−0.620	0.399	-	
8. Flourishing	0.111	−0.025	−0.282	−0.355	−0.466	0.665	0.472	-

*N* = 499. Gender was coded 0 = male and 1 = female. Exact confidence intervals and *p*-values for hypothesis-relevant coefficients are reported in the text; significance stars are intentionally omitted.

**Table 4:** Ordinal WLSMV confirmatory factor analysis fit indices.

Model	*χ* ^2^	df	CFI	TLI	RMSEA	SRMR
FoMO one factor	1094.13	27	0.689	0.585	0.335	0.124
FoMO two correlated factors	304.32	26	0.941	0.918	0.149	0.044
Loneliness one factor	131.43	9	0.955	0.925	0.147	0.036
Belonging one factor	2164.70	54	0.605	0.517	0.268	0.163
Belonging two correlated factors	319.23	53	0.934	0.917	0.111	0.043
Flourishing one factor	281.67	20	0.915	0.881	0.144	0.050
Nonrelational flourishing five items	89.28	5	0.944	0.888	0.168	0.040
Belonging–flourishing merged	3574.95	170	0.631	0.587	0.187	0.128
Acceptance, rejection, flourishing separate	862.53	167	0.898	0.884	0.099	0.048
Acceptance–flourishing merged; rejection separate	1427.69	169	0.825	0.804	0.129	0.068
Combined five-factor model	3497.20	550	0.776	0.757	0.116	0.082
Combined six-factor model	1683.53	545	0.878	0.867	0.086	0.050

*N* = 499. Models were estimated in R 4.6.0 under webR using lavaan 0.7–2.3166, with ordered items, theta parameterization, standardized latent variances, and the WLSMV specification. The *χ*^2^ values are scaled statistics; lavaan reports the estimator as DWLS with a scaled-shifted test. All models converged and passed the post-estimation admissibility check, and all scaled *χ*^2^ tests were statistically significant, *p* < 0.001. RMSEA confidence intervals for the focal models are reported in the Results. Standardized factor loadings and indicator *R*^2^ values for the combined six-factor model are presented in Supplementary Table S1, and selected local-fit diagnostics are presented in Supplementary Table S2. Fit indices from the covariate-adjusted structural SEMs were interpreted separately because those models evaluated different conditional covariance structures and were assessed against different baseline models.

**Table 5:** Hierarchical regression model summary for flourishing with HC3-robust block tests.

Block	Variables added	*R* ^2^	Adjusted *R*^2^	Δ*R*^2^	HC3 Wald *F*	df	*p*
1	Age and gender	0.012	0.009	0.012	4.12	(2, 496)	0.017
2	Personal and social FoMO	0.138	0.131	0.125	35.97	(2, 494)	<0.001
3	Loneliness	0.271	0.264	0.134	72.14	(1, 493)	<0.001
4	Acceptance/inclusion and rejection/exclusion (rev.)	0.512	0.505	0.241	43.82	(2, 491)	<0.001

*N* = 499. Gender was coded 0 = male and 1 = female. FoMO, fear of missing out; Δ*R*^2^ change in explained variance associated with the variables entered at each block. The Δ*R*^2^ values were calculated using unrounded *R*^2^ estimates and are reported as descriptive increments in explained variance. The HC3 Wald *F* tests evaluate the joint null hypothesis that the coefficients newly entered at each block equal zero in the corresponding expanded model. The final seven-predictor model yielded HC3 Wald *F*(7, 491) = 53.99, *p* < 0.001.

**Table 6:** Final hierarchical regression model predicting flourishing.

Predictor	B	HC3 SE	*β*	95% CI	*p*	Partial *r*	Semi-*r*	VIF
Age	−0.003	0.010	−0.008	[−0.022, 0.017]	0.789	−0.011	−0.008	1.06
Female	0.060	0.094	0.024	[−0.124, 0.244]	0.521	0.033	0.023	1.08
Personal FoMO	−0.039	0.033	−0.047	[−0.104, 0.027]	0.249	−0.053	−0.037	1.63
Social FoMO	−0.061	0.050	−0.061	[−0.160, 0.038]	0.230	−0.066	−0.046	1.71
Loneliness	−0.173	0.072	−0.136	[−0.314, −0.032]	0.016	−0.146	−0.103	1.75
Acceptance/inclusion	0.481	0.061	0.526	[0.360, 0.601]	< 0.001	0.550	0.461	1.30
Rejection/exclusion (rev.)	0.110	0.048	0.138	[0.015, 0.204]	0.023	0.145	0.102	1.84

= 499. Gender was coded 0 = male and 1 = female. FoMO, fear of missing out; B, unstandardized regression coefficient; *β*, standardized regression coefficient; CI, confidence interval; HC3 SE, heteroskedasticity-consistent standard error. Confidence intervals and *p*-values were based on HC3 robust standard errors. Reverse-scored rejection/exclusion was coded so that higher scores indicated lower perceived rejection/exclusion.

**Table 7:** LMG relative-importance decomposition of the final model.

Predictor	LMG *R*^2^ contribution	% of model *R*^2^
Acceptance/inclusion	0.291	56.9
Loneliness	0.080	15.6
Rejection/exclusion (reversed)	0.079	15.5
Social FoMO	0.038	7.3
Personal FoMO	0.021	4.0
Age	0.002	0.5
Gender indicator	0.001	0.1

*N* = 499. LMG contributions sum to the final model *R*^2^; totals may differ slightly because of rounding.

### Measurement models and reliability

All WLSMV models converged without inadmissible solutions; however, overall model fit was mixed. For FoMO, the correlated Personal and Social FoMO model (CFI = 0.941, TLI = 0.918, RMSEA = 0.149, 95% CI [0.133, 0.166], SRMR = 0.044) provided a substantially better fit than the one-factor model (CFI = 0.689, TLI = 0.585, RMSEA = 0.335, SRMR = 0.124). Nevertheless, the elevated RMSEA and suboptimal TLI indicated that the preferred two-factor model did not demonstrate uniformly satisfactory fit. Similarly, the correlated acceptance/inclusion and reverse-scored rejection/exclusion model for belonging (CFI = 0.934, TLI = 0.917, RMSEA = 0.111, 95% CI [0.101, 0.121], SRMR = 0.043) fit substantially better than the one-factor alternative (CFI = 0.605, TLI = 0.517, RMSEA = 0.268, SRMR = 0.163), but its elevated RMSEA indicated remaining model misfit. The one-factor ULS-6 model yielded CFI = 0.955, TLI = 0.925, RMSEA = 0.147, and SRMR = 0.036, whereas the one-factor flourishing model yielded CFI = 0.915, TLI = 0.881, RMSEA = 0.144, and SRMR = 0.050. Thus, the low SRMR values and the comparative advantages of the specified models provided partial support for the intended scoring structures. However, given the elevated RMSEA values and several suboptimal CFI and TLI values, the factor structures were not interpreted as definitively confirmed in the present sample.

The combined six-factor model—Personal FoMO, Social FoMO, loneliness, acceptance/inclusion, reverse-scored rejection/exclusion, and flourishing—fit better (CFI = 0.878, TLI = 0.867, RMSEA = 0.086, 95% CI [0.082, 0.090], SRMR = 0.050) than the five-factor model in which the two belonging facets were collapsed (CFI = 0.776, TLI = 0.757, RMSEA = 0.116, SRMR = 0.082). However, the six-factor model also showed imperfect global fit, particularly in its CFI, TLI, and RMSEA values. Accordingly, the six constructs were retained as theoretically specified and comparatively preferable to the collapsed alternative rather than interpreted as having a fully confirmed measurement structure.

All standardized loadings in the combined six-factor model were positive and statistically significant and ranged from 0.635 to 0.934; indicator *R*^2^ values ranged from 0.403 to 0.871 (Supplementary Table S1). Thus, the imperfect global fit did not appear to arise from weak primary loadings. Local-fit diagnostics identified a limited number of larger residual associations: the largest absolute residual correlation was 0.170, 39 of the 595 item pairs exceeded |0.10|, and four were at least |0.15|. The largest modification indices were concentrated around ULS-6 item 4 and several within-scale item pairs (Supplementary Table S2), suggesting localized cross-loading or residual-dependence patterns rather than uniformly weak measurement. The local diagnostics were treated descriptively, and the theoretically specified model was retained without data-driven respecification.

The structural SEMs yielded more favorable global fit indices than the stand-alone six-factor CFA. However, this difference should not be interpreted as evidence that the measurement-model limitations were resolved. The structural models included observed covariates and structural regression relations in addition to the six-factor measurement specification. Consequently, they evaluated a different conditional covariance structure, had different degrees of freedom, and were assessed against different baseline models. CFI, TLI, and RMSEA values are therefore not directly comparable across these specifications. Consistent with this interpretation, SRMR was essentially unchanged across the six-factor CFA (0.050), direct SEM (0.051), and indirect SEM (0.051). The structural estimates were accordingly interpreted as exploratory conditional associations, while the mixed fit of the stand-alone CFA remained a limitation of the measurement model.

To evaluate the factorial distinctiveness of belonging and flourishing, the three-factor model separating acceptance/inclusion, reverse-scored rejection/exclusion, and flourishing (CFI = 0.898, TLI = 0.884, RMSEA = 0.099, SRMR = 0.048) was compared with two alternative specifications. It fit better than both the single-factor model merging all items (CFI = 0.631, TLI = 0.587, RMSEA = 0.187, SRMR = 0.128) and the model merging acceptance/inclusion with flourishing while retaining rejection/exclusion as a separate factor (CFI = 0.825, TLI = 0.804, RMSEA = 0.129, SRMR = 0.068). The polychoric HTMT value for belonging and flourishing was 0.783, and the latent correlations of flourishing with acceptance/inclusion and reverse-scored rejection/exclusion were 0.773 and 0.593, respectively. These estimates were more consistent with related but distinct constructs than with a single merged factor. However, because the separate three-factor model also showed suboptimal CFI, TLI, and RMSEA values, the results do not establish unambiguous discriminant validity and do not eliminate conceptual or item-content overlap. Alpha values ranged from 0.891 to 0.912 and omega values from 0.918 to 0.951 across the primary scales and facets.

### Zero-order associations

Personal FoMO was positively associated with loneliness, *r* = 0.318, 95% CI [0.237, 0.395], and negatively associated with acceptance/inclusion, *r* = −0.222, 95% CI [−0.304, −0.137], reverse-scored rejection/exclusion, *r* = −0.336, 95% CI [−0.412, −0.256], and flourishing, *r* = −0.282, 95% CI [−0.360, −0.199], all *p* < 0.001. Social FoMO showed the same pattern with loneliness, *r* = 0.302, 95% CI [0.220, 0.379], acceptance/inclusion, *r* = −0.328, 95% CI [−0.404, −0.247], reverse-scored rejection/exclusion, *r* = −0.418, 95% CI [−0.488, −0.343], and flourishing, *r* = −0.355, 95% CI [−0.429, −0.275], all *p*-values < 0.001.

Loneliness was negatively associated with flourishing, *r* = −0.466, 95% CI [−0.532, −0.394], whereas acceptance/inclusion, *r* = 0.665, 95% CI [0.613, 0.712], and reverse-scored rejection/exclusion, *r* = 0.472, 95% CI [0.400, 0.537], were positively associated with flourishing (all *p* values < 0.001). Dependent-correlation comparisons showed that Social FoMO was more strongly related than Personal FoMO to acceptance/inclusion, *t*(496) = 2.72, *p* = 0.007, and reverse-scored rejection/exclusion, *t*(496) = 2.20, *p* = 0.028. The Personal and Social FoMO correlations did not differ significantly for loneliness, *t*(496) = 0.42, *p* = 0.675, or flourishing, *t*(496) = 1.91, *p* = 0.057. These findings supported H1 at the facet level: Personal and Social FoMO were positively associated with loneliness and negatively associated with both belonging facets. H2 was also supported at the zero-order level: loneliness was negatively associated with flourishing, whereas both belonging facets were positively associated with flourishing.

### Hierarchical regression and predictor importance

Age and gender accounted for 1.2% of flourishing variance, *R*^2^ = 0.012, and their joint HC3-robust contribution was statistically significant, Wald *F*(2, 496) = 4.12, *p* = 0.017. Adding Personal and Social FoMO increased explained variance by 12.5%, Δ*R*^2^ = 0.125, HC3 Wald *F*(2, 494) = 35.97, *p* < 0.001. Loneliness added 13.4%, Δ*R*^2^ = 0.134, HC3 Wald *F*(1, 493) = 72.14, *p* < 0.001, and the two belonging facets added a further 24.1%, Δ*R*^2^ = 0.241, HC3 Wald *F*(2, 491) = 43.82, *p* < 0.001. The final model explained 51.2% of flourishing variance, *R*^2^ = 0.512, adjusted *R*^2^ = 0.505, and the HC3-robust omnibus test was statistically significant, Wald *F*(7, 491) = 53.99, *p* < 0.001.

Using HC3 robust inference, loneliness had a small negative unique association with flourishing, B = −0.173, *β* = −0.136, 95% CI [−0.314, −0.032], *p* = 0.016. Acceptance/inclusion had the largest positive coefficient, B = 0.481, *β* = 0.526, 95% CI [0.360, 0.601], *p* < 0.001, and reverse-scored rejection/exclusion had a smaller positive coefficient, B = 0.110, *β* = 0.138, 95% CI [0.015, 0.204], *p* = 0.023. The Personal FoMO, Social FoMO, age, and gender coefficients were not statistically significant in the final model. These findings supported H3: loneliness and both belonging facets showed statistically significant unique associations with flourishing after adjustment for age, gender, and the two FoMO facets. However, the rejection/exclusion coefficient was not statistically significant when the explicitly relational Flourishing Scale items were omitted, indicating that this association was less robust than the loneliness and acceptance/inclusion associations.

The LMG decomposition attributed 56.9% of the model *R*^2^ to acceptance/inclusion, 15.6% to loneliness, 15.5% to reverse-scored rejection/exclusion, 7.3% to Social FoMO, 4.0% to Personal FoMO, and less than 1% jointly to age and gender. The LMG estimates describe the relative allocation of explained variance among correlated predictors.

### Exploratory latent direct-association model

The exploratory six-factor latent direct-association model yielded scaled *χ*^2^(613) = 1833.57, *p* < 0.001, CFI = 0.964, TLI = 0.966, RMSEA = 0.063, 95% CI [0.060, 0.067], and SRMR = 0.051. Because this model included observed covariates and structural regressions, these indices were interpreted within the specified model and do not supersede the mixed fit observed for the stand-alone measurement models. The standardized coefficients broadly reproduced the observed-score pattern: loneliness was negatively associated with flourishing, *β* = −0.118, 95% CI [−0.208, −0.028], *p* = 0.010; acceptance/inclusion was positively associated with flourishing, *β* = 0.595, 95% CI [0.524, 0.666], *p* < 0.001; and reverse-scored rejection/exclusion showed a smaller positive association, *β* = 0.137, 95% CI [0.057, 0.218], *p* = 0.001. The coefficients for Personal FoMO, *β* = −0.054, *p* = 0.319, and Social FoMO, *β* = −0.037, *p* = 0.520, were not statistically significant. Age showed a small positive coefficient, *β* = 0.111, *p* = 0.021, whereas gender was not statistically significant, *β* = −0.053, *p* = 0.238.

### Regression diagnostics and sensitivity analyses

Multicollinearity was not problematic: tolerance ranged from 0.544 to 0.943 and VIFs from 1.06 to 1.84. The Breusch-Pagan test indicated heteroskedasticity, LM(7) = 84.94, *p* < 0.001; therefore, HC3 standard errors were used. A joint HC3 test of quadratic terms did not provide strong evidence of nonlinearity, *F*(5, 486) = 1.07, *p* = 0.376. The Durbin-Watson value was 1.88; because the records were unordered cross-sectional observations, independence was evaluated primarily from the sampling design rather than from observation order. Residual skewness was 0.047 and excess kurtosis was 1.075. The maximum Cook’s D was 0.120, below 1.00; 36 observations exceeded 4/N, 36 exceeded the 2p/N leverage screen, and six had absolute studentized residuals above 3. These flags were examined in sensitivity analyses rather than treated as automatic exclusion criteria.

Retaining the two duplicate-pattern records (*N* = 501) and restricting the sample to ages 18–29 (*n* = 477) produced the same substantive pattern. When relational Flourishing Scale items 2, 4, and 8 were omitted, acceptance/inclusion remained positively associated with the outcome, *β* = 0.469, *p* < 0.001, and loneliness remained negatively associated, *β* = −0.168, *p* = 0.005; reverse-scored rejection/exclusion was no longer significant, *β* = 0.091, *p* = 0.117. Shared relational item content therefore does not fully account for the acceptance/inclusion and loneliness associations, whereas the attenuation of the rejection/exclusion coefficient indicates that this association may have been more sensitive to content overlap. After excluding the 36 Cook’s D screening cases, the loneliness and belonging results remained, while Personal FoMO became a small significant coefficient, *β* = −0.078, *p* = 0.026. The null Personal FoMO coefficient was therefore less stable than the loneliness and acceptance/inclusion coefficients.

### Exploratory cross-sectional indirect associations

The exploratory latent indirect-association model yielded scaled *χ*^2^(607) = 1901.33, *p* < 0.001, CFI = 0.962, TLI = 0.963, RMSEA = 0.065, 95% CI [0.062, 0.069], and SRMR = 0.051. These global fit indices were interpreted within the covariate-adjusted structural specification and do not negate the mixed fit of the stand-alone measurement model. Personal and Social FoMO, together with age and gender, accounted for 16.1% of the variance in latent loneliness, 19.4% of the variance in acceptance/inclusion, and 29.0% of the variance in reverse-scored rejection/exclusion. The full model explained 65.1% of the variance in latent flourishing. The constituent standardized structural paths, covariate paths, residual correlations, and endogenous *R*^2^ values are reported in Table 8 and the focal structural coefficients are displayed in Figure 1. The specific indirect, total indirect, direct, and total associations are reported in Table 9. The standardized total indirect association was −0.120 for Personal FoMO, 95% CI [−0.212, −0.028], *p* = 0.010, and −0.269 for Social FoMO, 95% CI [−0.360, −0.178], *p* < 0.001. Personal FoMO showed small indirect associations through loneliness, *β* = −0.029, 95% CI [−0.055, −0.003], *p* = 0.029, and reverse-scored rejection/exclusion, *β* = −0.024, 95% CI [−0.046, −0.001], *p* = 0.038; its acceptance/inclusion path included zero, *β* = −0.068, *p* = 0.059. For Social FoMO, the acceptance/inclusion path was the largest, *β* = −0.198, 95% CI [−0.277, −0.118], *p* < 0.001, followed by reverse-scored rejection/exclusion, *β* = −0.053, 95% CI [−0.090, −0.016], *p* = 0.005; the loneliness path included zero, *β* = −0.018, *p* = 0.110. The direct Personal and Social FoMO coefficients remained nonsignificant. The observed-score 5,000-bootstrap sensitivity model yielded the same broad pattern, with total indirect estimates of −0.073 and −0.222, respectively. These exploratory cross-sectional indirect estimates quantify model-implied covariance patterns only. Whether FoMO precedes loneliness or belonging in time, or operates through them in any causal sense, is a separate question that this cross-sectional design cannot answer. The sensitivity analyses for the primary hierarchical regression are summarized in Table 10.

**Table 8:** Constituent standardized paths and explained variance in the exploratory latent indirect-association model.

Panel A: Standardized structural paths				
Endogenous variable	Predictor	*β*	95% CI	*p*
Loneliness	Personal FoMO	0.240	[0.103, 0.377]	<0.001
Loneliness	Social FoMO	0.153	[0.014, 0.291]	0.031
Loneliness	Age	−0.121	[−0.215, −0.026]	0.012
Loneliness	Female	0.098	[0.010, 0.186]	0.029
Acceptance/inclusion	Personal FoMO	−0.112	[−0.229, 0.005]	0.060
Acceptance/inclusion	Social FoMO	−0.329	[−0.448, −0.209]	<0.001
Acceptance/inclusion	Age	0.133	[0.038, 0.228]	0.006
Acceptance/inclusion	Female	−0.038	[−0.126, 0.049]	0.389
Rejection/exclusion (rev.)	Personal FoMO	−0.173	[−0.300, −0.046]	0.008
Rejection/exclusion (rev.)	Social FoMO	−0.382	[−0.506, −0.257]	<0.001
Rejection/exclusion (rev.)	Age	0.128	[0.032, 0.224]	0.009
Rejection/exclusion (rev.)	Female	−0.046	[−0.136, 0.043]	0.312
Flourishing	Personal FoMO	−0.054	[−0.161, 0.053]	0.319
Flourishing	Social FoMO	−0.037	[−0.149, 0.075]	0.520
Flourishing	Loneliness	−0.120	[−0.211, −0.028]	0.010
Flourishing	Acceptance/inclusion	0.601	[0.530, 0.672]	< 0.001
Flourishing	Rejection/exclusion (rev.)	0.138	[0.057, 0.220]	< 0.001
Flourishing	Age	−0.002	[−0.065, 0.062]	0.963
Flourishing	Female	−0.011	[−0.072, 0.049]	0.710

Panel B: Explained variance	
Endogenous variable	*R* ^2^
Loneliness	0.161
Acceptance/inclusion	0.194
Rejection/exclusion (rev.)	0.290
Flourishing	0.651

Panel C: Latent correlation and residual correlations				
Variable 1	Variable 2	Standardized estimate	95% CI	*p*
Loneliness	Acceptance/inclusion	−0.422	[−0.491, −0.353]	<0.001
Loneliness	Rejection/exclusion (rev.)	−0.648	[−0.704, −0.592]	<0.001
Acceptance/inclusion	Rejection/exclusion (rev.)	0.403	[0.337, 0.470]	<0.001
Personal FoMO	Social FoMO	0.716	[0.676, 0.757]	<0.001

*N* = 499. WLSMV estimation with ordered items, theta parameterization, and standardized latent variances. Gender was coded 0 = male and 1 = female. Personal and Social FoMO were freely correlated. Residual correlations among loneliness, acceptance/inclusion, and reverse-scored rejection/exclusion were freely estimated because these concurrent variables were not assumed to be conditionally independent. Coefficients and confidence intervals are standardized delta-method estimates. The model is exploratory and cross-sectional.

**Figure 1:**
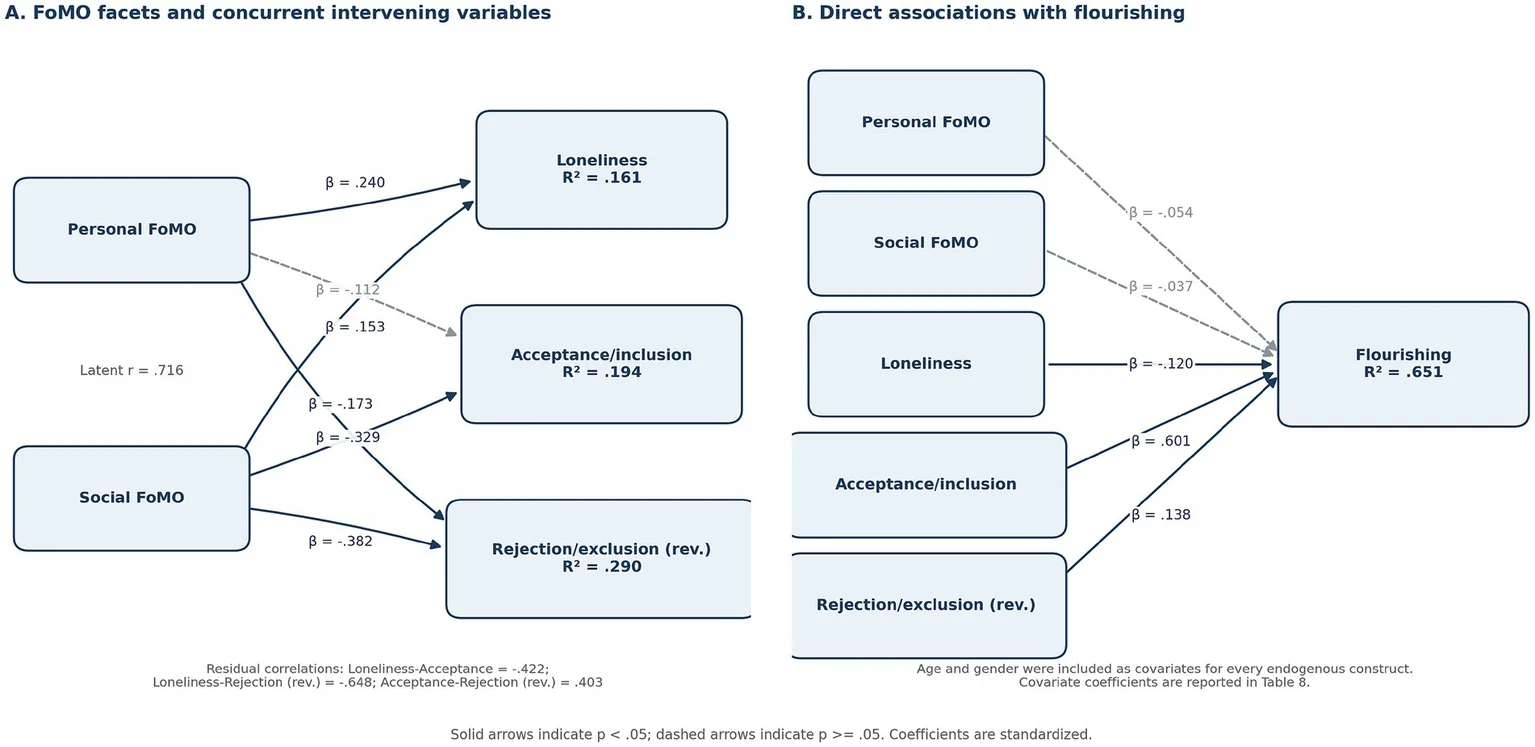
Exploratory latent cross-sectional indirect-association model. Standardized focal coefficients are shown. Solid arrows indicate *p* < 0.05 and dashed arrows indicate *p* ≥ 0.05. Age and gender were included as covariates for each endogenous latent construct; their coefficients are reported in Table 8. Residual correlations among loneliness, acceptance/inclusion, and reverse-scored rejection/exclusion were freely estimated. The figure represents exploratory cross-sectional model-implied associations and does not indicate causal mediation or temporal ordering.

**Table 9:** Exploratory latent cross-sectional indirect, direct, and total associations.

Effect	Standardized estimate	Delta-method 95% CI	*p*
Personal FoMO via loneliness	−0.029	[−0.055, −0.003]	0.029
Personal FoMO via acceptance/inclusion	−0.068	[−0.138, 0.003]	0.059
Personal FoMO via rejection/exclusion (rev.)	−0.024	[−0.046, −0.001]	0.038
Personal FoMO: total indirect	−0.120	[−0.212, −0.028]	0.010
Personal FoMO: direct	−0.054	[−0.161, 0.053]	0.319
Personal FoMO: total association	−0.174	[−0.299, −0.050]	0.006
Social FoMO via loneliness	−0.018	[−0.041, 0.004]	0.110
Social FoMO via acceptance/inclusion	−0.198	[−0.277, −0.118]	<0.001
Social FoMO via rejection/exclusion (rev.)	−0.053	[−0.090, −0.016]	0.005
Social FoMO: total indirect	−0.269	[−0.360, −0.178]	<0.001
Social FoMO: direct	−0.037	[−0.149, 0.075]	0.520
Social FoMO: total association	−0.306	[−0.434, −0.177]	<0.001

*N* = 499. The latent model was estimated using WLSMV with ordered items, theta parameterization, and standardized latent variances. Age and gender were included as observed covariates, with gender coded 0 = male and 1 = female. Standardized estimates and confidence intervals were obtained using the delta method. Total indirect associations represent the sums of the three specific indirect associations, and total associations represent the sums of the direct and total indirect associations. CI, confidence interval; FoMO, fear of missing out. The estimates represent exploratory cross-sectional statistical associations and should not be interpreted as causal mediation or temporal pathways.

**Table 10:** Sensitivity analyses for the primary hierarchical regression.

Analysis	*n*	*R* ^2^	Summary
All 501 records retained	501	0.513	Loneliness and both belonging facets significant; FoMO facets not significant
Age 18–29 only	477	0.511	Same substantive pattern as the primary model
Nonrelational flourishing outcome	499	0.427	Acceptance and loneliness significant; rejection/exclusion not significant
Cook’s D > 4/N cases omitted	463	0.641	Core loneliness/belonging pattern retained; Personal FoMO became small and significant

The influence-based deletion model is a robustness check only; it does not redefine the analytic sample.

## Discussion

### Principal findings and hypothesis evaluation

This study examined the concurrent associations among Personal FoMO, Social FoMO, loneliness, two facets of belonging, and flourishing in university students. H1 was supported: both FoMO facets were positively associated with loneliness and negatively associated with acceptance/inclusion and reverse-scored rejection/exclusion. H2 was also supported at the zero-order level: loneliness was negatively associated with flourishing, whereas both belonging facets were positively associated with flourishing. In the hierarchical regression, loneliness and both belonging facets retained statistically significant unique associations with flourishing after adjustment for age, gender, and the FoMO facets, supporting H3. However, the rejection/exclusion coefficient was not statistically significant when the explicitly relational Flourishing Scale items were omitted, indicating that this association was less robust than those of loneliness and acceptance/inclusion.

The final model explained approximately half of the observed variance in flourishing. Acceptance/inclusion had the largest standardized coefficient and LMG relative-importance contribution, loneliness retained a smaller negative coefficient, and reverse-scored rejection/exclusion retained a smaller positive coefficient. Personal and Social FoMO were negatively associated with flourishing at the zero-order level but did not retain statistically significant unique coefficients in the full model, indicating that their associations with flourishing were largely shared with loneliness and belonging in the present sample. The larger acceptance/inclusion coefficient indicates a stronger concurrent association within this model but does not establish temporal, causal, or psychological priority.

### FoMO in the shared relational domain

The negative zero-order associations of both FoMO facets with flourishing are consistent with university-student research associating FoMO with adverse well-being-related functioning (Hayran and Anik, 2021) and situating it within a broader network involving well-being, loneliness, and problematic digital engagement (O'Brien et al., 2023). Additional facet-level comparisons indicated that Social FoMO was more strongly related than Personal FoMO to both belonging facets, whereas the two FoMO–flourishing correlations did not differ statistically. Neither FoMO facet retained a significant unique coefficient in the full model, indicating that their zero-order associations with flourishing overlapped statistically with loneliness, belonging, and the other predictors. The nonsignificant adjusted coefficients do not invalidate the bivariate associations; rather, they show that neither FoMO facet accounted for unique variation in flourishing after all other predictors were included.

This pattern also accords with longitudinal evidence that questions simple one-way accounts. Groenestein et al. (2025) found consistent between-person associations of FoMO with psychological need satisfaction and well-being but only limited and nonreciprocal within-person evidence. Bonfanti et al. (2023) reported a within-person path from loneliness to subsequent FoMO, whereas the reverse path was not supported. Similarly, Wang et al. (2025) found that baseline loneliness was positively associated with later trait- and state-FoMO. Guo et al. (2026) likewise specified T1 loneliness before T2 FoMO and T3 social networking site addiction in a three-wave longitudinal mediation model, while noting that reverse and bidirectional alternatives were not tested. Overall, these studies do not support treating FoMO as uniformly antecedent to loneliness; the observed ordering varies across models and remains compatible with reciprocal and unmeasured third-variable explanations.

### Loneliness, belonging, and flourishing

Loneliness was associated with both belonging facets but was not redundant with them. It retained a small negative coefficient after both belonging facets and the remaining predictors were included, consistent with evidence from Turkish university students showing that loneliness was negatively associated with psychological well-being measured using the Flourishing Scale (Cinalioğlu and Gazioğlu, 2022). Acceptance/inclusion had the largest standardized coefficient and LMG relative-importance contribution in the present model. This facet-specific finding converges with broader European evidence: across five universities in five countries, institutional belonging was positively associated with well-being at every institution and negatively associated with loneliness, although the strength of the latter association varied across sites (Pleschová et al., 2026). A meta-analysis of 56 higher-education studies similarly reported a moderate-to-strong negative association between belonging and loneliness ( r=−.48 ; Dost, 2026). Evidence from Türkiye also indicated a positive adjusted association between social connectedness and life satisfaction in a convenience sample of 810 students recruited from several universities on the European side of Istanbul (Güllü et al., 2026). Social connectedness and life satisfaction are not equivalent to belonging and flourishing, respectively; this finding therefore provides contextual convergence rather than a direct replication. In the present sample, acceptance/inclusion remained positively associated with flourishing after adjustment for all other predictors.

The two belonging facets should not be combined into a single score without stronger psychometric justification. The correlated two-factor model fit substantially better than the one-factor alternative, although its absolute fit remained mixed. The facets also differed in the sensitivity analysis: acceptance/inclusion remained positively associated with the reduced flourishing score after the three explicitly relational items were excluded, whereas reverse-scored rejection/exclusion did not. Positive inclusion and the absence of rejection or exclusion may therefore contain partly different statistical information. This interpretation remains provisional, however, because the observed difference may reflect substantive distinctions, item wording, or reverse-scoring effects and requires replication in independent samples.

### Construct overlap and robustness

The strong association between acceptance/inclusion and flourishing warranted explicit evaluation of potential criterion contamination arising from overlapping relational content, because the Flourishing Scale intentionally includes supportive relationships, social respect, and contributing to others’ happiness as components of flourishing (Diener et al., 2010). The three-factor model separating acceptance/inclusion, reverse-scored rejection/exclusion, and flourishing fit better than the alternatives that merged these constructs. The separation of the two belonging facets was also consistent with the original development and Turkish validation studies, both of which identified Acceptance/Inclusion and Rejection/Exclusion as two highly correlated factors of general belongingness (Malone et al., 2012; Duru, 2015). The HTMT and latent-correlation estimates in the present study were more consistent with closely related but nonidentical constructs than with a single undifferentiated construct. However, neither the separate three-factor model nor the combined six-factor measurement model achieved uniformly satisfactory global fit. These analyses therefore provide comparative evidence against collapsing belonging and flourishing into a single construct, but they do not establish unequivocal discriminant validity.

Acceptance/inclusion and loneliness remained statistically associated with the reduced flourishing score after the three explicitly relational Flourishing Scale items were omitted. Lu et al. (2024) reported a conceptually similar sensitivity analysis: after removing 14 social-relationship items from the 84-item Psychological Well-Being Scale, perceived social support remained positively associated with the reduced well-being measure after adjustment for sex, age, and subjective socioeconomic status. Perceived social support is not equivalent to belonging, and Lu et al. used a different measure of psychological well-being. Nevertheless, the two studies provide convergent evidence that associations between positive relational appraisals and well-being cannot be explained entirely by explicitly relational outcome items. These sensitivity analyses reduce, but do not eliminate, the possibility that shared relational content contributed to the primary associations. In both the original and Turkish versions of the General Belongingness Scale, the two factors are also aligned with positively and negatively worded items (Malone et al., 2012; Duru, 2015). Consequently, the attenuation of the reverse-scored rejection/exclusion coefficient suggests that the two facets should not be treated as predictively interchangeable, but the present analysis cannot determine whether their differential robustness reflects substantive differences, wording-related variance, or both.

The principal loneliness and belonging results were retained when all 501 non-straightlining records were analyzed, when the sample was restricted to participants aged 18–29 years, and when observations exceeding the Cook’s distance screening threshold were omitted. Personal FoMO, however, changed from a nonsignificant coefficient in the primary model to a small statistically significant coefficient after the Cook’s distance cases were excluded. Because exceeding the 4/N screening threshold identifies potentially influential observations rather than invalid data, the full N=499 analysis remains primary. The Personal FoMO coefficient should therefore be interpreted as sensitive to influence-screening decisions rather than as a robust unique association.

### Theoretical and cultural interpretation

Self-determination theory and the belongingness hypothesis help organize the observed associations around the relational domain, but the data do not constitute a test of all propositions of either theory. Autonomy, competence, need satisfaction, and behavioral mechanisms were not measured. Similarly, although Türkiye is often discussed in cross-cultural research, collectivism and self-construal were not assessed here. The role of cultural orientation should be examined in comparative research rather than inferred from the current coefficients.

The exploratory indirect models indicated that loneliness and belonging accounted statistically for part of the FoMO–flourishing covariance, particularly through the Social FoMO–acceptance/inclusion pathway. Longitudinal or experimental designs are needed to evaluate temporal and causal mechanisms.

## Limitations and future directions

The cross-sectional design prevents conclusions about temporal precedence, causal direction, or mediation. All constructs were assessed by self-report within the same survey, which may have increased common-method covariance. The indirect-association models should therefore be understood as descriptions of model-implied covariance rather than evidence that FoMO operates through loneliness or belonging. Convenience and snowball recruitment began in one department and continued through participants’ online networks. Institutional and programme affiliations were not collected, so the number, identity, and representativeness of the participating universities cannot be established. Recruitment-chain identifiers and referral links were also not retained; consequently, possible dependence within recruitment chains and homophily among referred participants could not be assessed statistically. Such unmeasured network clustering may have affected the precision and generalizability of the estimates. Online recruitment may also have favored students who were more digitally engaged. In addition, 76.4% of the analytic sample was female. This imbalance limits generalizability to male students and reduced the precision available for gender-specific analyses; measurement invariance and group-specific structural models were therefore not examined. The findings apply to university students aged 18–30 years and should not be generalized to all adults or to the full period of emerging adulthood.

Measurement-model fit was mixed. The correlated FoMO and belonging models fit substantially better than their one-factor alternatives, but several models showed elevated RMSEA values and suboptimal CFI or TLI values. The combined six-factor model and the three-factor belonging–flourishing model also failed to show uniformly satisfactory global fit. Although the original ULS-6 was evaluated in older adults, its Turkish adaptation was subsequently examined in an adult sample. Internal consistency was high in the current university sample, but the elevated RMSEA warrants caution regarding its unidimensional structure. The factor structures and discriminant validity of the measures should be replicated in independent university samples before stronger measurement claims are made. Although the primary factor loadings were strong, the local-fit diagnostics identified several potentially sample-specific cross-loading and residual-dependence patterns. The more favorable fit indices of the covariate-adjusted structural models do not remove these concerns because those models evaluated a different conditional covariance structure and were assessed against different baseline models. Replication studies should therefore evaluate both global and local fit and test any theoretically defensible item-level respecifications in independent samples. The online survey did not include a CAPTCHA, dedicated attention-check item, or unique participant identifier. Data screening identified three extreme straightlining records and an exact three-record response pattern, but repeated participation could not be confirmed or completely ruled out. The main findings were unchanged when all 501 non-straightlining records were retained. The absence of responses in the category indicating more than 5 h of daily social-media use also warrants caution. Although daily duration and checking frequency measured different aspects of use, the self-reported duration distribution may not have captured actual exposure accurately.

The study and analysis plan were not preregistered. Facet-level comparisons, relative-importance estimates, latent indirect associations, and sensitivity analyses should therefore be replicated using prospectively specified hypotheses, scoring rules, measurement models, and analysis plans. The primary model also did not include every collected demographic, health, and digital-use variable. No *a priori* adjustment set had been specified, and several of these variables could plausibly represent consequences, mediators, or colliders rather than confounders. Future research should preregister a causal model, recruit students across documented institutions and programmes, use more precise measures of digital behavior, and examine longitudinal within-person associations in more gender-balanced samples.

## Conclusion

Personal and Social FoMO, loneliness, and both belonging facets were associated with flourishing at the bivariate level. In the full model, loneliness and the two belonging facets retained unique coefficients, whereas the FoMO facets did not. Acceptance/inclusion showed the largest association, although the rejection/exclusion coefficient was less robust when relational flourishing items were excluded. These findings highlight the shared relational variance among the constructs and warrant replication in longitudinal studies.

## Data Availability

The raw data supporting the conclusions of this article will be made available by the authors, without undue reservation.

## References

[ref1] Baumeister R. F. Leary M. R. (1995). The need to belong: desire for interpersonal attachments as a fundamental human motivation. Psychol. Bull. 117, 497–529. doi: 10.1037/0033-2909.117.3.497, 7777651

[ref2] Berlingieri F. Colagrossi M. Mauri C. (2023). Loneliness and Social Connectedness: Insights From a New EU-wide Survey. Brussels: European Commission, Joint Research Centre.

[ref3] Bonfanti R. C. Salerno L. Ruggieri S. Lo Coco G. (2023). Interplay between fear of missing out and loneliness: a three-wave panel study during the COVID-19 pandemic. Cyberpsychol. Behav. Soc. Netw. 26, 447–452. doi: 10.1089/cyber.2022.0182, 37057987

[ref4] Çelik F. Özkara B. Y. (2022). Gelişmeleri Kaçırma Korkusu (FoMO) Ölçeği: Sosyal medya bağlamına uyarlanması ve psikometrik özelliklerinin sınanması. Psikol. Calismalari 42, 71–103. doi: 10.26650/SP2021-838539

[ref5] Cinalioğlu E. A. Gazioğlu E. İ. (2022). Psychological well-being in emerging adulthood: the role of loneliness, social support, and sibling relationships in Turkey. Can. J. Fam. Youth 14, 102–123. doi: 10.29173/cjfy29753

[ref6] d’Hombres B. Kovacic M. Schnepf S. V. Blaskó Z. (2024). Loneliness and Social Media Use in the European Union. Luxembourg: European Commission, Joint Research Centre.

[ref7] Deci E. L. Ryan R. M. (2000). The 'what' and 'why' of goal pursuits: human needs and the self-determination of behavior. Psychol. Inq. 11, 227–268. doi: 10.1207/S15327965PLI1104_01

[ref8] Diener E. Wirtz D. Tov W. Kim-Prieto C. Choi D. W. Oishi S. . (2010). New well-being measures: short scales to assess flourishing and positive and negative feelings. Soc. Indic. Res. 97, 143–156. doi: 10.1007/s11205-009-9493-y

[ref9] Dost G. (2025). Student well-being: the impact of belonging, COVID-19 pandemic related student stress, loneliness, and academic anxiety. Front. Psychol. 16:1481328. doi: 10.3389/fpsyg.2025.1481328, 40357492 PMC12066278

[ref10] Dost G. (2026). Belongingness and loneliness in higher education: a meta-analysis of pre- and post-pandemic trends. Front. Psychol. 16:1625957. doi: 10.3389/fpsyg.2025.1625957, 41705169 PMC12908175

[ref11] Duru E. (2015). Genel Aidiyet Ölçeğinin psikometrik özellikleri: Geçerlik ve güvenirlik çalışması. Turk. Psikol. Dan. Reh. Derg. 5, 37–47.

[ref12] Eurostat (2025). 97% of Young People in the EU Use the Internet Daily. Luxembourg, Eurostat: European Commission. Available online at: https://ec.europa.eu/eurostat/web/products-eurostat-news/w/edn-20250715-1 (Accessed June 9, 2026).

[ref13] Groenestein E. Willemsen L. van Koningsbruggen G. M. Kerkhof P. (2025). Fear of missing out and social media use: a three-wave longitudinal study on the interplay with psychological need satisfaction and psychological well-being. New Media Soc. 27, 4205–4227. doi: 10.1177/14614448241235935

[ref14] Güllü S. Şahinler Y. Genç A. Bozyiğit E. Biçer Baikoğlu S. Genç N. . (2026). The relationship among university students’ recreational flow experience, psychological resilience, social connectedness, and life satisfaction. Front. Psychol. 17:1774124. doi: 10.3389/fpsyg.2026.1774124, 42111569 PMC13152732

[ref15] Guo J. Guo Y. Jia Z. Zhang M. (2026). The relationship between loneliness and social networking site addiction among Chinese college students: the roles of fear of missing out and the imaginary audience. Front. Psychol. 17:1792932. doi: 10.3389/fpsyg.2026.1792932, 42058292 PMC13121311

[ref16] Hayran C. Anik L. (2021). Well-being and fear of missing out (FoMO) on digital content in the time of COVID-19: a correlational analysis among university students. Int. J. Environ. Res. Public Health 18:1974. doi: 10.3390/ijerph18041974, 33670639 PMC7922067

[ref17] Henseler J. Ringle C. M. Sarstedt M. (2015). A new criterion for assessing discriminant validity in variance-based structural equation modeling. J. Acad. Mark. Sci. 43, 115–135. doi: 10.1007/s11747-014-0403-8

[ref18] Holzer J. Lüftenegger M. Korlat S. Pelikan E. Salmela-Aro K. Spiel C. . (2021). Higher education in times of COVID-19: university students' basic need satisfaction, self-regulated learning, and well-being. AERA Open 7:23328584211003164. doi: 10.1177/23328584211003164, 34192126 PMC7961159

[ref19] Lu H. Song Y. Wang X. Liu J. (2024). The neural correlates of perceived social support and its relationship to psychological well-being. Front. Behav. Neurosci. 17:1295668. doi: 10.3389/fnbeh.2023.1295668, 38259632 PMC10800560

[ref20] MacKinnon J. G. White H. (1985). Some heteroskedasticity-consistent covariance matrix estimators with improved finite sample properties. J. Econom. 29, 305–325. doi: 10.1016/0304-4076(85)90158-7

[ref21] Malone G. P. Pillow D. R. Osman A. (2012). The general belongingness scale (GBS): assessing achieved belongingness. Pers. Individ. Differ. 52, 311–316. doi: 10.1016/j.paid.2011.10.027

[ref22] Neto F. (2014). Psychometric analysis of the short-form UCLA loneliness scale (ULS-6) in older adults. Eur. J. Ageing 11, 313–319. doi: 10.1007/s10433-014-0312-1, 28804337 PMC5549168

[ref23] O'Brien O. Sumich A. Baguley T. Kuss D. J. (2023). A partial correlation network indicates links between wellbeing, loneliness, FOMO and problematic internet use in university students. Behav. Inf. Technol. 42, 2717–2734. doi: 10.1080/0144929X.2022.2142845

[ref24] Pleschová G. Hajdúk M. Simon E. Januška J. Zemandl E. Nyikes N. . (2026). Student belonging and well-being in higher education: insights from five European universities. Policy Futures in Education Advance Online Publication. London: SAGE Publications. doi: 10.1177/14782103261436155

[ref25] Przybylski A. K. Murayama K. DeHaan C. R. Gladwell V. (2013). Motivational, emotional, and behavioral correlates of fear of missing out. Comput. Hum. Behav. 29, 1841–1848. doi: 10.1016/j.chb.2013.02.014

[ref26] Santini Z. I. Pisinger V. S. C. Nielsen L. Madsen K. R. Nelausen M. K. Koyanagi A. . (2021). Social disconnectedness, loneliness, and mental health among adolescents in Danish high schools: a nationwide cross-sectional study. Front. Behav. Neurosci. 15:632906. doi: 10.3389/fnbeh.2021.632906, 33927599 PMC8078177

[ref27] Sarıçam H. (2023). COVID-19 sonrası yetişkinlerde yalnızlık: UCLA Yalnızlık Ölçeği Kısa Formunun (UYÖKF-6) psikometrik özellikleri. Iğdır Üniversitesi Sosyal Bilimler Dergisi 32, 1–17. doi: 10.54600/igdirsosbilder.1066242

[ref28] Telef B. B. (2013). Psikolojik İyi Oluş Ölçeği: Türkçeye uyarlama, geçerlik ve güvenirlik çalışması. Hacettepe Üniversitesi Eğitim Fakültesi Dergisi 28, 374–384.

[ref29] Uyaroğlu A. K. Ergin E. Tosun A. S. Erdem Ö. (2022). A cross-sectional study of social media addiction and social and emotional loneliness in university students in Turkey. Perspect. Psychiatr. Care 58, 2263–2271. doi: 10.1111/ppc.13056 35152424

[ref30] Vitale E. M. Smith A. S. (2022). Neurobiology of loneliness, isolation, and loss: integrating human and animal perspectives. Front. Behav. Neurosci. 16:846315. doi: 10.3389/fnbeh.2022.846315, 35464141 PMC9029604

[ref31] von Elm E. Altman D. G. Egger M. Pocock S. J. Gøtzsche P. C. Vandenbroucke J. P. (2007). The strengthening the reporting of observational studies in epidemiology (STROBE) statement: guidelines for reporting observational studies. PLoS Med. 4:e296. doi: 10.1371/journal.pmed.0040296, 17941714 PMC2020495

[ref32] Wang Y. Sun Y. Li T. (2025). The longitudinal relationship between loneliness and problematic social networking site use in college students: the mediating role of trait- and state-fear of missing out. Front. Psychol. 16:1477239. doi: 10.3389/fpsyg.2025.1477239, 40177036 PMC11961881

[ref33] Zhang Z. Jiménez F. R. Cicala J. E. (2020). Fear of missing out scale: a self-concept perspective. Psychol. Mark. 37, 1619–1634. doi: 10.1002/mar.21406

