# Supplementary material for: Flourishing in university students: cross-sectional associations with personal and social FoMO, loneliness, and belonging
Source: Front Behav Neurosci. 2026 Sep 29;20:1914187. doi: 10.3389/fnbeh.2026.1914187 (PMC13658611; doi:10.3389/fnbeh.2026.1914187)
Supplement: Supplementary file 1 [file Data_Sheet_1.pdf]

Supplementary Table S1. Combined Six-Factor CFA Standardized Loadings and Indicator R^2

Factor	Indicator	Standardized loading	Indicator R^2	p
Personal FoMO	PFoMO item 1	.829	.687	< .001
Personal FoMO	PFoMO item 2	.842	.709	< .001
Personal FoMO	PFoMO item 3	.915	.837	< .001
Personal FoMO	PFoMO item 4	.900	.810	< .001
Personal FoMO	PFoMO item 5	.916	.839	< .001
Social FoMO	SFoMO item 1	.913	.833	< .001
Social FoMO	SFoMO item 2	.904	.817	< .001
Social FoMO	SFoMO item 3	.890	.792	< .001
Social FoMO	SFoMO item 4	.934	.871	< .001
Loneliness	ULS item 1	.837	.700	< .001
Loneliness	ULS item 2	.842	.709	< .001
Loneliness	ULS item 3	.846	.715	< .001
Loneliness	ULS item 4	.896	.803	< .001
Loneliness	ULS item 5	.755	.570	< .001
Loneliness	ULS item 6	.789	.623	< .001
Acceptance/inclusion	GBS item 1	.709	.503	< .001
Acceptance/inclusion	GBS item 2	.849	.721	< .001
Acceptance/inclusion	GBS item 3	.873	.762	< .001
Acceptance/inclusion	GBS item 4	.859	.738	< .001
Acceptance/inclusion	GBS item 5	.734	.538	< .001
Acceptance/inclusion	GBS item 6	.872	.761	< .001
Rejection/exclusion (rev.)	GBS item 7	.712	.506	< .001
Rejection/exclusion (rev.)	GBS item 8	.889	.789	< .001
Rejection/exclusion (rev.)	GBS item 9	.893	.797	< .001
Rejection/exclusion (rev.)	GBS item 10	.794	.631	< .001
Rejection/exclusion (rev.)	GBS item 11	.838	.702	< .001
Rejection/exclusion (rev.)	GBS item 12	.916	.838	< .001
Flourishing	FS item 1	.795	.632	< .001
Flourishing	FS item 2	.866	.750	< .001
Flourishing	FS item 3	.739	.546	< .001
Flourishing	FS item 4	.635	.403	< .001
Flourishing	FS item 5	.683	.467	< .001
Flourishing	FS item 6	.787	.619	< .001
Flourishing	FS item 7	.719	.517	< .001
Flourishing	FS item 8	.842	.709	< .001

Note. N = 499. Estimates are from the combined six-factor ordinal CFA estimated with WLSMV, ordered items, theta parameterization, and standardized latent variances. PFoMO = Personal FoMO; SFoMO = Social FoMO; ULS = six-item loneliness scale; GBS = General Belongingness Scale; FS = Flourishing Scale. All loadings were positive and statistically significant.

Supplementary Table S2. Selected Local-Fit Diagnostics for the Combined Six-Factor CFA

Panel A. Largest absolute raw residual correlations

Indicator 1	Indicator 2	Residual correlation	Absolute residual
ULS item 2	FS item 4	.170	.170
SFoMO item 3	ULS item 4	.167	.167
ULS item 2	GBS item 7	.155	.155
ULS item 6	FS item 4	.152	.152
GBS item 3	GBS item 7	.149	.149
SFoMO item 2	ULS item 5	-.143	.143
SFoMO item 2	ULS item 6	-.142	.142
SFoMO item 4	ULS item 4	.141	.141
ULS item 1	FS item 4	.138	.138
ULS item 6	FS item 6	.136	.136
ULS item 3	GBS item 7	.133	.133
ULS item 4	ULS item 6	-.133	.133
SFoMO item 2	FS item 3	.133	.133
FS item 6	FS item 7	.132	.132
GBS item 2	GBS item 7	.128	.128
ULS item 4	FS item 8	-.127	.127
GBS item 11	FS item 5	-.126	.126
FS item 1	FS item 4	-.126	.126
ULS item 3	FS item 4	.124	.124
GBS item 10	FS item 5	-.123	.123

Panel B. Largest modification indices

Candidate parameter	MI	EPC	Standardized EPC
Rejection/exclusion (rev.) =~ ULS item 4	159.70	-1.077	-.478
Social FoMO =~ ULS item 4	133.97	.667	.296
Flourishing =~ ULS item 4	125.09	-.684	-.303
Acceptance/inclusion =~ ULS item 4	106.56	-.607	-.269
Personal FoMO =~ ULS item 4	84.37	.530	.235
Loneliness =~ FS item 4	50.68	.289	.223
Rejection/exclusion (rev.) =~ FS item 8	50.36	.337	.182
Social FoMO =~ PFoMO item 5	44.82	.638	.256

Candidate parameter	MI	EPC	Standardized EPC
FS item 6 ~~ FS item 7	43.11	.383	.383
ULS item 1 ~~ ULS item 2	42.61	.460	.460
PFoMO item 5 ~~ SFoMO item 1	41.93	.868	.868
ULS item 5 ~~ ULS item 6	41.18	.382	.382
Loneliness =~ FS item 8	37.52	-.302	-.163
Personal FoMO =~ SFoMO item 1	37.05	.590	.241
Loneliness =~ GBS item 3	35.10	-.295	-.144
Flourishing =~ ULS item 6	33.95	.249	.153
Rejection/exclusion (rev.) =~ FS item 5	33.11	-.217	-.158
Acceptance/inclusion =~ GBS item 7	33.04	.194	.136
Rejection/exclusion (rev.) =~ GBS item 3	29.66	.259	.126
Loneliness =~ FS item 2	29.42	-.287	-.144

Note. Raw residual correlations equal the observed polychoric correlation minus the model-implied correlation. MI = modification index; EPC = expected parameter change. Diagnostics are descriptive and post hoc. No candidate cross-loading or residual covariance was added to the final model because these modifications were not prespecified and require confirmation in an independent sample.
